# Supplementary material for: Acute Toxicity of the Dinoflagellate Amphidinium carterae on Early Life Stages of Zebrafish (Danio rerio)
Source: Toxics. 2023 Apr 13;11(4):370. doi: 10.3390/toxics11040370 (PMC10144361; doi:10.3390/toxics11040370)
Supplement: Supplementary file 1 [file toxics-11-00370-s001.zip › toxics-2303190-supplementary.pdf]

toxics

Supplementary materials

Acute Toxicity of the Dinoflagellate *Amphidinium carterae* on Early Life Stages of Zebrafish (*Danio rerio*)

Xiao Yang [1,2], Zhi Yan [1,3], Jingjing Chen [1,4], Derui Wang [1,5] and Ke Li [1,6,*]

Figure S1. Single mass composition analysis of amphidinol 18 **(A)**, amphidinol 19 **(B)** and amphidinol 2 **(C)**.

A

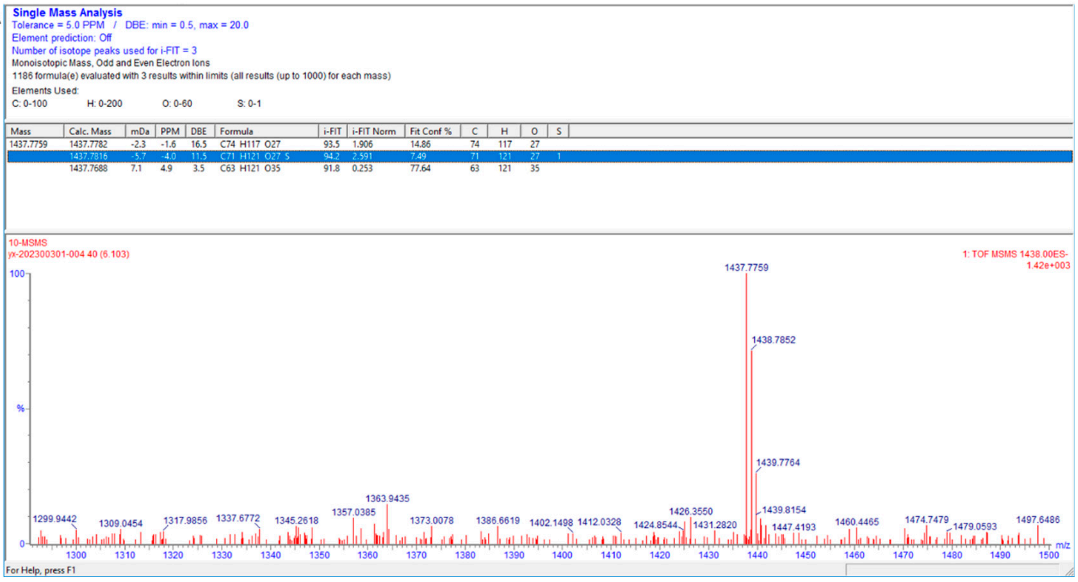

B

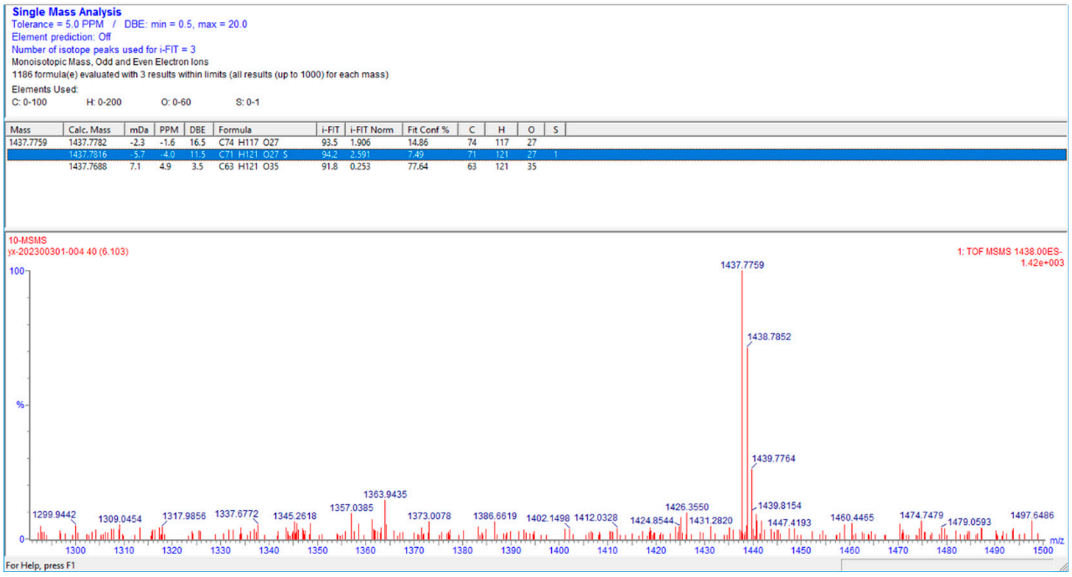

C

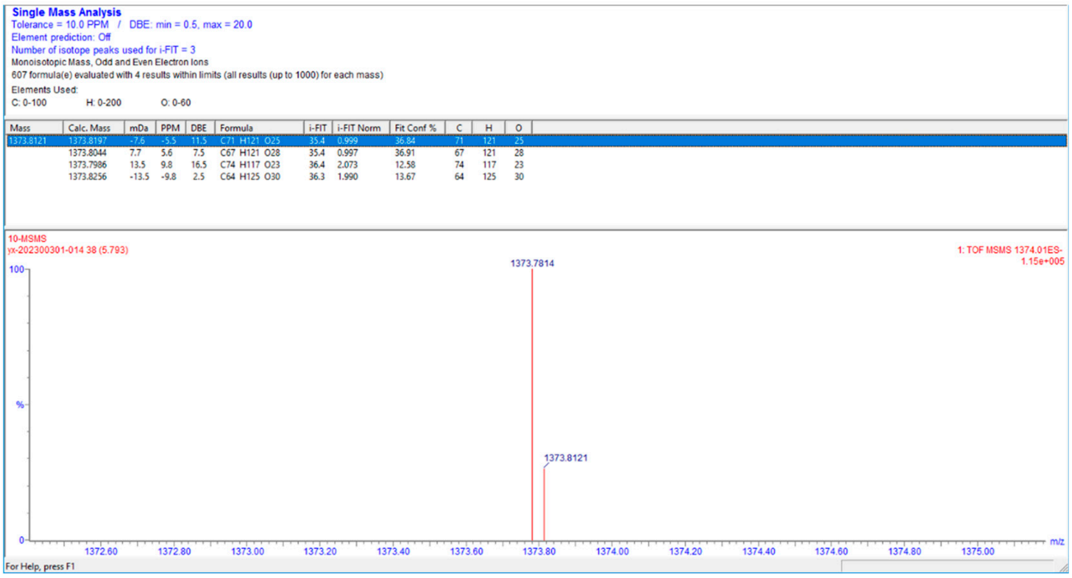

Mass	Calc. Mass	mDa	PPM	DBE	Formula	i-FIT	i-FIT Norm	Fit Conf %	C	H	O
1373.8121	1373.8197	-7.6	-5.5	11.5	C71 H121 O25	35.4	0.999	36.84	71	121	25
	1373.8044	7.7	5.6	7.5	C67 H121 O28	35.4	0.997	36.91	67	121	28
	1373.7986	13.5	9.8	16.5	C74 H117 O23	36.4	2.073	12.58	74	117	23
	1373.8256	-13.5	-9.8	2.5	C64 H125 O30	36.3	1.990	13.67	64	125	30
